# Influencing Mechanism of Job Satisfaction on Safety Behavior of New Generation of Construction Workers Based on Chinese Context: The Mediating Roles of Work Engagement and Safety Knowledge Sharing

**DOI:** 10.3390/ijerph17228361

**Published:** 2020-11-12

**Authors:** Guodong Ni, Yuanyuan Zhu, Ziyao Zhang, Yaning Qiao, Huaikun Li, Na Xu, Yongliang Deng, Zhenmin Yuan, Wenshun Wang

**Affiliations:** 1 School of Mechanics & Civil Engineering, China University of Mining and Technology, Xuzhou 221116, China; zhuyuanyuancumt@cumt.edu.cn (Y.Z.); zhangziyao@cumt.edu.cn (Z.Z.); yaning.qiao@cumt.edu.cn (Y.Q.); lihuaikun@cumt.edu.cn (H.L.); na_xu@yeah.net (N.X.); dylcumt@cumt.edu.cn (Y.D.); tbh171@cumt.edu.cn (Z.Y.); wangws@cumt.edu.cn (W.W.); 2 Research Center for Digitalized Construction and Knowledge Engineering, China University of Mining and Technology, Xuzhou 221116, China

**Keywords:** safety behavior, job satisfaction, work engagement, health and safety, construction worker

## Abstract

China’s construction industry developed rapidly and safety production has become a vital issue. Improving the safety behavior of construction workers is an important measure to effectively decrease construction safety accidents. At present, a New Generation of Construction Workers (NGCWs) born after 1980 has gradually become the main force of construction companies in China and the special group characteristics coming from the intergenerational difference may make them behave differently in safety-related activities, therefore, it is very important to study how to promote their safety behavior. This paper aimed to explore the influencing mechanism of job satisfaction on the safety behavior of NGCWs and examine the mediating role of safety knowledge sharing and work engagement. Confirmatory factor analysis and structural equation modeling analysis were applied to test the theoretical model. Empirical research results indicated that job satisfaction can effectively promote safety behavior through safety knowledge sharing and work engagement. Safety knowledge sharing plays a complete mediating role between job satisfaction and safety compliance behavior, as well as between job satisfaction and safety participation behavior. Moreover, work engagement plays a complete mediating role between job satisfaction and safety participation behavior, which can provide valuable management references for China’s construction companies to strengthen their safety behavior.

## 1. Introduction

The construction industry has made significant contributions to China’s economy. For a long time, the risk of high accident rates and mortality has been one of the greatest concerns of this industry [1,2]. According to previous studies, human error (such as unsafe behavior) is determined as one of the main causes of construction accidents [3,4]. Construction workers play a core role in behavioral safety and are the most important factor in safety management [5]. The safety/unsafe behavior of construction workers has attracted a fair amount of research attention in recent years [6,7]. Meanwhile, with China’s rapid urbanization, more and more migrant workers began to move into big cities, and a large part of them joined the construction industry [8,9]. Among them, the new generation of construction workers (NGCWs) are gradually drawing more and more attention in recent years [10,11]. Prior literature has revealed the unique roles of demographic factors like age and seniority in this domain, and cohort studies are specific to the group of young workers [12,13,14]. The previous research shows that compared with the older workers, young workers have a lower awareness of safety [13], higher occupational injury rates [15], and lower safety voice intentions [16]. Therefore, it is worthwhile to conduct in-depth investigations about the improvement of safety performance to a single population, especially for the younger generation [17].

In this study, the NGCWs are defined as laborers and skilled workers born after 1980 who are engaged in construction-related work in China. Most of them have rural household registration but no longer rely on farming for a living, and a small proportion of them have urban household registration. Compared with their predecessors, the NGCWs have obvious group characteristics similar to Chinese young “migrant” workers, such as more awareness of fairness and rights protection [18], better education [19], higher physical and spiritual pursuit [20], more individualism [19], and these are the more important differences than age and seniority between the NGCWs and the older construction workers. These intergenerational differences can have a significant impact on their ideas, attitudes and behaviors [21]. Formal controls (e.g., penalties) may be ineffective in eliciting desired behavioral changes for the improvement of safety behavior [22]. One explanation could be that traditional safety management overemphasizes workers’ compliance with various rules and regulations but neglects to guide and encourage workers’ willingness and enthusiasm in safety [5]. To this end, scholars have begun to investigate the antecedents of workers’ safety behavior (SB) in the context of construction, and many achievements have been gained. Many scholars have sought to identify the related distal contextual factors (e.g., transformational leadership, impact of the supervisor, safety climate and aging expectation) [1,7,23,24] and proximal individual differences (e.g., risk perception, personality traits, and safety knowledge) [24,25,26], general workers [1,7], older construction workers [24] and ethnic minority construction workers [23] were included in these studies. However, no study has examined the antecedents or promotion mechanisms underlying the SB among Chinese young construction workers, i.e., NGCWs. In addition, as an important factor that can reflect positive attitude and stimulate behavioral initiative [27], job satisfaction (JS) is less associated with the SB of construction workers. Previous sporadic clues point to a positive relationship between JS and construction workers’ safety perception [12,28], thus further investigation of the relationship between the JS and SB of construction workers is required. Therefore, this study aimed to fill the gaps in the construction safety literature by investigating the relationship and influence mechanisms between the JS and SB of NCGWs. 

## 2. Theoretical Background

### 2.1. Safety Behavior (SB)

The SB is considered an action taken by an individual to promote the health and safety of their own self and working environment [29]. It is widely agreed that SB contains two dimensions: safety compliance behavior (SCB) and safety participation behavior (SPB). The former refers to adhering to safety procedures and carrying out work in a safe manner with the feature of a frequently voluntary, while the latter means helping co-workers promoting the safety program within the workplace, demonstrating initiative, and making an effort in improving workplace safety with the feature of being generally mandated to do so [30]. Previous studies have revealed that the risks or shortcuts taken by workers, or non-compliance with safety rules and procedures, are frequently the major causes of accidents [29]. Conversely, workers’ compliance and participation in safety can help to improve safety concerns and the organization’s safety program, and play an active role in preventing and reducing injuries and accidents [31]. In view of the necessity to improve SB, scholars have carried out exploratory studies from different various perspectives and covered a wide range of topics. From the perspective of proximal individuals, individual personality factors like personality traits [26], safety knowledge and safety motivation [32], safety attitude, subjective norm, perceived behavioral control and behavioral habit [24,33] can significantly affect workers’ SB. In addition, individual perceptions or expectations of workplace safety, namely the psychological climate [34] and psychological contract [35,36] also have a positive impact on the implementation of the SB. From the perspective of a distal context, leadership and safety climate are main factors in in previous literature [23,37,38]. As workers are profound and frequently interactive personnel, the supervision behavior of supervisors has both direct and indirect influences on workers’ SB [1]. 

Management behavior on a construction site plays an important role in improving the safety performance of workers’ behavior [39]. When more transformational leadership styles are shown, construction workers can be encouraged to express safety concerns without fear of retaliation, and work safety is repeatedly reminded, so safety climate intervention measures should be more effective [7]. As the premise of safety performance, a safety climate is manifested as construction workers’ general understanding of the values and importance of safety in the organization [40]. Common values and customs, namely group norms, play an important role in shaping the SB of individual workers [6]. He et al. [37] summarized three ways in which safety climate affects the SB of construction workers: (1) directly; (2) through mediating variables, such as stress, safety knowledge, motivation, and intention; and (3) through moderating variables, such as project identity, site layout, and work arrangement. 

### 2.2. Job Satisfaction (JS)

As an emotion, JS involves a person’s overall evaluation of their work environment [41]. JS is generally considered as a key element of employees and has a close relationship with job performance [42]. Meanwhile, this kind of pleasant and positive emotions can directly promote employees’ in-role and extra-role behaviors, such as work engagement (WE) [43], knowledge sharing behavior [27] and organization citizenship behavior [44]. Given the importance of JS, researchers have paid close attention to the determinants of JS in different contexts. These factors include the characteristics related to work and the work environment (such as safety, promotion, participation in decision making, salary), training and learning opportunities, social support, and perceived pressure [27,45]. On the whole, there are complex combinations of influencing factors of the JS. A special case is that even if the level of balance between work and family is low, high levels of teamwork and high senses of organizational identification and commitment can also improve the JS [41]. In the construction industry, researchers have also begun to realize the important role of JS, and research on the topic of JS gradually increases. From top to bottom, the research objects involve project managers [46,47], project management personnel and technicians [27], foreign workers [48] and construction craft workers [49]. Research reveals the unique role of JS in the construction industry, which can promote project success [50] and knowledge sharing among project members [27] in complex project situations. Especially for safety in construction workplaces, JS can not only significantly affect workers’ perception of safety [12], but also have a strong relationship with safety climate creation (managers and organizations). Employees with high JS have a more positive view of organizations’ engagement in controlling safety practices [28].

Prior studies found that the JS of construction workers is affected by many factors, including job characteristics, safety priority, organizational effectiveness, rewards, resource adequacy, physical and mental health, relations with superiors and co-workers, and the fulfillment of higher order needs [49,51]. There are also significant differences in the JS among different construction worker groups [51]. With the differences in age, characteristics and interests of the NGCWs, specific research on JS should be considered. This is consistent with the viewpoint of Guglielmi et al. [52], that age differences among workers should be taken into account in order to design appropriate human resource practices to promote JS. There is evidence that age differences may affect the relationship between JS and perceived management commitment to safety at work. Young employees are weaker than older employees in terms of safety climate perception under the same level of the JS [28].

### 2.3. Safety Knowledge Sharing (SKS)

As an important part and stage of knowledge management, knowledge sharing means that individuals, teams and organizations share knowledge with other members in the form of activities in various ways [53]. According to the type of knowledge and the difficulty of expression, it can be divided into explicit knowledge sharing and tacit knowledge sharing [27]. Explicit knowledge in organizations is systematic and can be coded and communicated; conversely, tacit knowledge is subjective and difficult to express, capture and share, but it has huge potential value [54,55]. Explicit knowledge is definite such as templates, patents, reports and checklists, while tacit knowledge instantiates personal experience, personal beliefs, perspectives and values [56]. The inherent impression of the construction industry is labor-oriented, but knowledge-sharing behavior can be observed everywhere in this industry. For example, unskilled construction workers are often assigned to work with more experienced workers so that they can learn through observation and interaction [57]. Given the uniqueness and complexity of construction projects, it is impossible for people to copy best practices directly from the past but they can learn from the past [58]. Hence, it is very important to transfer or share accumulated knowledge among workers [59]. From the perspective of safety, the construction safety knowledge is in safety regulations, accident records, best practice, and safety experts’ experience. It is necessary to exchange experience and information containing valuable knowledge within and across departmental, organizational, and geographical boundaries [60,61]. Lack of construction safety information exchange and knowledge sharing may lead to accidents and reduce productivity at construction sites [62].

It is found that the SKS has a positive impact on workers’ safe work conduct, and the research of Nesheim and Gressgård also reveals that training, internal motivation, work autonomy and management support can affect the level of knowledge-sharing behavior [60]. The NGCWs generally have higher levels of education and better learning ability [19]. This allows for better adaptation for them to accept and understand safety-related knowledge in safety education and training. However, little is known about the antecedents of SKS of the NGCWs. 

### 2.4. Work Engagement (WE)

The work engagement (WE) is defined as a positive, fulfilling, work-related state of mind characterized by vigor, dedication, and absorption [63]. Vigor means investing with high levels of energy and willingness; dedication refers to active participation and enthusiasm; absorption is to fully and happily engage oneself at work [64]. In recent decades, WE has witnessed an explosion of research on job engagement, and one of the reasons for its popularity is that it can well predict the important outcomes of employees, teams and organizations [65,66]. As employees’ positive view of work, WE is considered a key indicator of organizational health and has a significant impact on employee performance, JS, turnover intention, customer satisfaction, organization success and company profitability [67]. In addition, WE is usually associated with positive organizational behavior [68]. Employees with high engagement have a more positive attitude towards their work and organization, pay more respect to their colleagues, help others heighten the work efficiency, improve their own work skills, and perform at a very active state, which contributes to improving the performance of their in-role and extra-role behaviors [67]. Lyu et al. [69] and Afsar et al. [70] have confirmed the opinion, which also shows that there is a positive association between engagement, organizational citizenship behavior and innovative work behavior. 

A previous study has noticed that age can affect employees’ WE [71]. Older workers have higher organizational identity, higher organizational loyalty, suppress negative information and events, and experience more positive emotions at work [52]. Compared with their predecessors, young workers usually have unique needs [71]. When workers’ expectations of need fulfillment are met, they may show higher engagement [72]. 

In brief, previous studies have begun to fix attention to the SB of construction workers. However, few studies have addressed whether the improvement of JS can promote the SB of construction workers. In addition, few studies have conducted specific investigations on the NGCWs, who are becoming the major labor force in China’s construction industry. To fill this research gap, the objectives of this paper are to explore the mechanism of improving the SB from the perspective of JS of the NGCWs, and use the WE and SKS as two moderating variables to establish a theoretical model. Based on this, this paper focuses on investigating the mediating roles of the WE and SKS in linking JS with workers’ SBs in order to seek a new solution for improving the SB of NGCWs in China.

## 3. Research Hypotheses and Theoretical Model

### 3.1. Hypotheses Development

#### 3.1.1. JS and SB

James et al. [73] have shown the SB happens more often in a comfortable climate and among people who are satisfied with their jobs. Employees with higher JS are more likely to perceive safety and safety climate at the construction sites [12,28]. As their perception increases, logically, they tend to reduce risk-taking or shortcut behaviors and display more SCBs. High satisfaction with work can motivate individuals to follow the rules and make them willing to pay extra attention to safety activities, which demonstrates that JS can influence SCB and SPB [74]. According to Probst and Brubakeer [75], individuals reporting higher levels of JS also report relatively higher levels of SB. On the contrary, the job dissatisfaction of workers can possibly lead to behaving more unsafely, thus causing more accidents and injuries [76]. The JS, as an emotional response [77], in some way, determines the performance in jobs and influences the outcome of SB at last. Therefore, the following hypotheses are proposed:

**Hypothesis 1a** **(H1a).** *JS has a significant positive effect on the SCB of NGCWs.*

**Hypothesis 1b** **(H1b).** *JS has a significant positive effect on the SPB of NGCWs.*

#### 3.1.2. JS, SKS and SB

The NGCWs tend to be more passionate and creative at work when they are satisfied with their job, which can motivate them to participate in SKS, such as sharing the news on construction accidents and tips for protecting themselves at work [78]. Studies in the non-construction area already proved that an obviously positive relationship existed between the JS and SKS [79]. For example, JS can significantly and positively affect the knowledge sharing behaviors of engineers in the electronic information industry [79]. Chumg et al. [80] researched on a virtual organization showing that when employees are fulfilled, they will increasingly contribute to both explicit knowledge sharing and tacit knowledge sharing at work. High levels of the JS individuals tend to be more pro-social to share knowledge with colleagues. Linking to social exchange theory, the behavior of sharing knowledge can be regarded as a reciprocation to the firm because of the JS gained from the organization [78]. Therefore, the following hypothesis is proposed:

**Hypothesis 2** **(H2).** *JS has a significant positive effect on the SKS of NGCWs.*

It is accepted that construction workers’ lack of safety knowledge is one of the causes for safety accidents [62]. Shin et al. indicated that among all the intervening variables like safety motivation and affective commitment, safety knowledge is the strongest direct effect on SB [40]. Similarly, Mohamadfam et al. [81] found that safety knowledge is one of the best predictors of SB. Safety knowledge management, therefore, is of vital importance. As the core link in knowledge management, knowledge sharing can be an effective way to diffuse knowledge, especially in a situation where safety training is insufficient at the construction site. Unsafe behavior can be rectified or even prevented through wide and full SKS [60]. Research reveals that knowledge sharing behavior further improves the quality of staff’s knowledge, their safety participation inclination and compliance with occupational safety regulations [82]. Therefore, the following hypotheses are proposed:

**Hypothesis 3a** **(H3a).** *SKS has a significant positive effect on the SCB of NGCWs.*

**Hypothesis 3b** **(H3b).** *SKS has a significant positive effect on the SPB of NGCWs.*

After analyzing the relationship between the JS and SKS, SKS and SB, it is clear that SKS can act as a mediator between JS and SB, although none of the research has proven the same thing. Logically, the JS enhances construction workers’ willingness to share safety knowledge [79], which leads to quick safety knowledge acquisition among construction workers, and safety knowledge is one of the best predictors of SB [81]. It is reasonable to surmise the mediator role that SKS plays. Therefore, the following hypotheses are proposed:

**Hypothesis 4a** **(H4a).** *SKS mediates the influence relationship between JS and SCB.*

**Hypothesis 4b** **(H4b).** *SKS mediates the influence relationship between JS and SPB.*

#### 3.1.3. JS, WE and SB

According to social exchange theory, the SB and WE can be regarded as the product of a reciprocal exchange activity between new generation workers and the organization. Higher levels of JS mean higher levels of WE. Present research proves that a close relationship exists between JS and WE. The improvement of quality and feeling of personal work life can result in greater engagement and professionalism [83]. If individuals’ JS improves, their WE will increase [84]. 

The intimate relationship between WE and SB has been verified by researchers through empirical studies [85,86]. According to Rich et al. [86], workers with high engagement levels are more likely to work with higher intensity, focus on role responsibilities, and become emotionally tied to their work and thus willingly obey safety regulations at work. Individuals can gain a larger resource repertoire when going through engagement at work, which makes them tend to improve workplace safety and allocate resources to prevent their colleagues from safety accidents [85]. In a report, for engaged workers, it cost USD 63 to cover a safety incident, and as for non-engaged workers, the number was USD 392 [87]. Gallup [88] has reported that business units with a low level of engagement level went through 62% more safety incidents compared with those with a high engagement level. Accordingly, accident rates can be predicted by the worker’s engagement levels [89]. In summary, it is clear that the JS, WE and SB have a connection with each other. Therefore, the following hypotheses are proposed:

**Hypothesis 5** **(H5).** *JS has a significant positive effect on the WE of NGCWs.*

**Hypothesis 6a** **(H6a).** *WE has a significant positive effect on the SCB of NGCWs.*

**Hypothesis 6b** **(H6b).** *WE has a significant positive effect on the SPB of NGCWs.*

**Hypothesis 7a** **(H7a).** *WE mediates the influence relationship between JS and SCB.*

**Hypothesis 7b** **(H7b).** *WE mediates the influence relationship between JS and SPB.*

### 3.2. Theoretical Model

**Figure 1:**
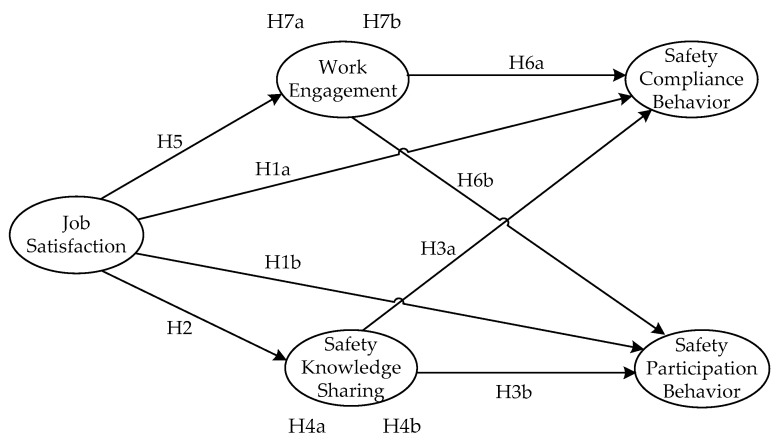
Theoretical model.

Based on the hypotheses proposed above, the theoretical model of this research was constructed as below in Figure 1, which reflects the expected influencing relationships and improvement paths among the research variables JS, SKS, WE, and SB. According to this theoretical model, and through the scientific empirical research method, this paper will explore the improving mechanism of SB about the NGCWs from the perspective of JS, and focus on investigating the important mediating roles of the WE and SKS in linking JS with SB for the purpose of seeking a new solution to improve the SB of NGCWs in China.

## 4. Method

### 4.1. Development of Measurement

All scales of this study were drawn or adapted from the existing literature in order to ensure the scientificity and reliability of the research tools. Many empirical types of research on SB have utilized Neal and Griffin’s measurement scale, dividing SB into SCB and SPB [90]. Therefore, the scale of SB was measured from SCB and SPB with 12 items, which were adapted from Vinodkumar et al. [91], Neal et al. [90,92], and Shin et al. [40]. For the scale of JS with a total of 24 items, this study referenced and modified from the scale of Spector [93], Davis [94], and Luan [95] from five dimensions i.e., satisfaction with pay (SP), satisfaction with co-workers (SC), satisfaction with work environment (SE), satisfaction with leaders (SL) and satisfaction with work itself (SW). According to Bock et al. [96], Wang et al. [97,98], Lin [99] and Casimir et al. [100], the measurement scale of SKS were drawn with two dimensions which included explicit safety knowledge sharing (ESKS) and tacit safety knowledge sharing (TSKS) and consisted of 10 items. The scale of WE with a total of 15 items was adopted from Schaufeli et al. [63] and Saks [101] with three dimensions including job involvement (JI), organizational identity (OI), and work value (WV). A five-point Likert scale was applied to measure all items. The details of the measurement scale can be seen in Appendix A Table A1. 

### 4.2. Sample and Data Collection

In this paper, the authors adopted a structured questionnaire survey method to collect the data and test the hypotheses. The target population of this paper are the NGCWs (below 40 years old) on the construction sites in China. The survey was conducted in 25 construction companies and the involving construction projects are located in 16 provinces and municipalities in China, which are Jiangsu, Sichuan, Henan, Shandong, Beijing, Fujian, Guangdong, Hunan, Ningxia, Shanxi, Shaanxi, Shanghai, Tianjin, Zhejiang, Chongqing and Hebei. The survey was conducted from 12 December 2019 to 16 January 2020. During the process, 800 questionnaires were distributed to a number of frontline construction workers both online (450) and offline (350) with the help of the human resources department or the construction project managers on sites. To help participants factually and precisely report, they were given a brief survey invitation that explained the survey’s academic purpose and provided a brief explanation of safety behaviors with certain examples. Finally, a total of 532 questionnaires (259 offline and 273 online) were answered, which gave a response rate of 66.5%. Excluding the questionnaires that do not meet the requirements (such as being older than 40 years old, filling the questionnaire in less than two minutes, giving the same evaluation to each item, and uncompleted questionnaires), a number of 368 valid responses were finally collected, which gave an effective response rate of 69.17 percent.

The sample structure is shown in Table 1 which presents a reasonable distribution of gender, age, marriage status, educational level, seniority, working hours in a day and the average monthly income. The majority of the sample were men with a percent of 93.2. The sample proportion of junior high school, certificate or associate’s degree, senior high school and junior college and above were 39.1%, 29.1%, 19.3% and 9.5%, respectively, which reflects relatively better the educational background of the NGCWs. Most of them had a work experience of 6 to 10 years. The sample whose working hours were eight to ten hours in a day took a proportion of 82.3%, and the difference of average monthly income was comparatively significant.

### 4.3. Data Analysis Methods

In this research, the data analysis methods mainly included confirmatory factor analysis (CFA) and structural equation modeling (SEM). The statistical analysis software of Statistical Product and Service Solutions 22.0 (IBM Corporation, Armonk, NY, USA) and Analysis of Moment Structures (AMOS) 21.0 (IBM Corporation, Armonk, NY, USA) were used, respectively. Using the maximum likelihood method, the CFA was performed to test whether all the measurement variables properly reflected their latent variables and whether the data fit in the theoretical measurement model well, also to confirm the validity of the constructs and research model [5]. The SEM was used to test the hypotheses and conduct a path analysis [102]. In addition, the bootstrapping method was adopted to verify the mediating role of the research variables by testing the significance of indirect effects which can avoid the problems caused by asymmetric and non-normal sampling distributions [103].

## 5. Results

### 5.1. CFA

The CFA was carried out to confirm the validity of the overall measurement including convergent validity and discriminant validity. According to Fornell and Larcker [104], convergent validity can be assessed by the factor loading (FL), composite reliability (CR), and average variance extracted (AVE). Among them, FL should exceed 0.6 [105], CR should exceed 0.8 [106], and AVE should be more than 0.5 [107]. In addition, the coefficients of Cronbach’s alpha were generated to assess the construct reliability, which should be higher than 0.7 [97]. The variables, constructs, the measurement items and reliability index (Cronbach’s alpha) and convergent validity indexes (FL, CR and AVE, respectively) are presented in Table 2. The Cronbach’s alpha of each construct is greater than 0.7, the FL of each item is greater than 0.6, the CR and AVE of all the constructs are higher than 0.8 and 0.5, respectively, which means all indexes reach the corresponding standards. Therefore, the results indicate that the model sufficiently meets the convergent validity and reliability criteria.

Discriminant validity represents the extent to which two conceptually similar concepts differ and can be tested by the criterion that the square root of the AVE of each latent variable from its indicators is greater than the correlation coefficients between the same construct and any other construct [59]. The means, standard deviations (SDs) and correlation coefficients among variables are shown in Table 3. The results show that most of the diagonal elements are basically higher than their respective off-diagonal elements, which indicates that the measurement model has desired discriminant validity. In addition, all variables are significantly and positively related thanks to the positive correlation coefficients between them as can be seen in Table 3, supporting the close relationship among research variables. Furthermore, the collinearity diagnostics were conducted using SPSS22.0 to assess the collinearity among all the measured variables. The results show that the maximal value of variance inflation factor (VIF) is 4.786, which is far below the recommended cut off value of 10. All values of tolerance (TOL) are smaller than 0.1, suggesting that multicollinearity does not exist between the measured variables.

According to Hair et al. [108], this study assessed the measurement model fit to verify if the measurement item presented a good fit to the data by evaluating:

- Absolute fit measures, including observed normed χ^2^ (χ^2^/df), root-mean square residual (RMR), goodness-of-fit index (GFI) and root mean square error of approximation (RMSEA);
- Incremental fit measures, including normed fit index (NFI), incremental fit index (IFI), tacker-lew is index (TLI) or non-normed fit index (NNFI), adjusted goodness-of-fit index (AGFI) and comparative fit index (CFI);
- Parsimonious fit measures, including parsimony goodness-of-fit index (PGFI), parsimony normed fit index (PNFI) and parsimony comparative fit index (PCFI).

The values of the above three categories of fitting indexes and the recommended cutoff values are presented in Table 4, which indicates that the fit indices meet ideal levels [1,105,106], hence the survey data can support the theoretical model and it is suitable for testing the research hypotheses. 

Overall, the results showed that this study had adequate validity and reliability and the problem of multiple collinearity did not exist. Moreover, the research model can properly fit in the obtained data.

### 5.2. SEM Analysis and Hypotheses Testing

**Figure 2:**
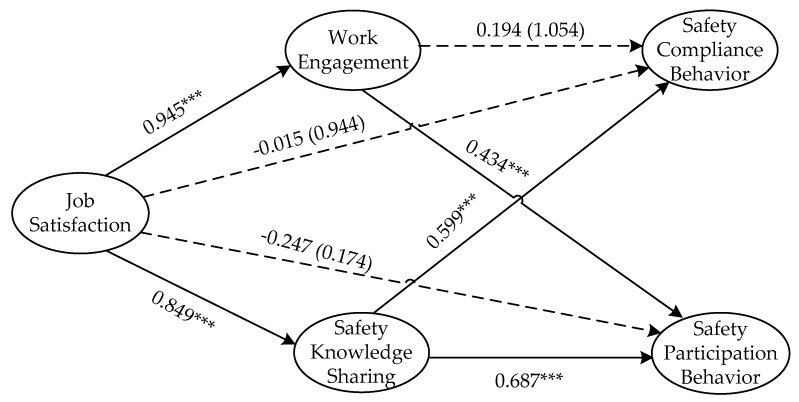
Research model and results of the hypothesis test. Note: the values on the lines are the path coefficients. The value in brackets is *p*. The solid lines and dashed lines indicate passed paths and rejected paths, respectively. ***, *p* < 0.001.

The SEM is a hybrid of the measurement model and the structural model. The former represents the hypothesized relationships among latent variables and their indicators; the latter is the path model connecting the independent and dependent variable and the hybrid model is able to integrate factor analysis and path analysis at the same time [102]. SEM was performed in this study to provide support for research hypotheses and establish a path analysis using AMOS 22.0. The critical ratio (C.R.) and *p* value were employed as two indices to evaluate the significance of the hypotheses. To be statistically significant (*p* < 0.05), the value of C.R. should be greater than 1.96 [59]. The results are shown in Figure 2 and Table 5.

Figure 2 and Table 5 show that, the effects of JS on SKS (JS–SKS, 0.849, *p* < 0.001), SKS on SCB and SPB (SKS–SCB, 0.599, *p* < 0.001; SKS–SPB, 0.687, *p* < 0.001), JS on WE (JS–WE, 0.945, *p* < 0.001), and WE on SPB (WE-SPB, 0.434, *p* < 0.001) are all significant. Therefore, H2, H3a, H3b, H5 and H6b are supported and pass the test. While the effects of JS on SCB and SPB (JS–SCB, −0.015, *p* = 0.944; JS–SPB, −0.247, *p* = 0.174), and WE on SCB (WE–SCB, 0.194, *p* = 0.292) are not significant, hence, H1a, H1b, H6a and H7a are all rejected and cannot pass the test.

To further verify whether SKS and WE both mediate the relationship between JS and SB, the bootstrapping method [103] was adopted. There are two steps to complete the process. The first step is to verify that the total indirect effect exists and the next step is to test that the specific indirect effect exists [109]. Following the related recommendations of MacKinnon et al. [110], if the confidence interval of bias-corrected (BC) and percentile (PC) does not have 0 included in both steps, then the mediating effect exists. Due to the failure of the path from WE to SCB, only paths referring to the mediation of JS–SKS–SCB, JS–SKS–SPB and JS–WE–SPB were tested using bootstrapping. The results of total mediating effect test from JS to SCB and SPB are shown in Table 6. The lower limit of the BC confidence interval is 0.580 and the upper limit is 0.752 with 0 excluded and the PC confidence intervals of 0.585 and 0.755, respectively, which indicates that the total indirect effect exists between the JS and SCB. Similarly, the total indirect effect exists between JS and SPB as well.

PRODCLIN2 developed by MacKinnon was used to testify the specific indirect effect [103], and the results were presented in Table 7. It can be seen that the lower and upper limits of the confidence interval of the specific indirect effect of JS on SPB through SKS are 0.416 and 0.769, respectively, excluding 0 and JS on SCB are 0.418 and 0.602, respectively, which illustrates that SKS mediates the influence effect of JS on SPB and SCB, and the total indirect effect are 0.993 and 0.692, respectively, therefore, H4a and H4b are supported. The similar conclusion can be drawn that WE plays a mediating role between JS and SPB, which supports H7b. 

## 6. Discussion

### 6.1. Summary of Findings

This study was conducted to provide a comprehensive understanding of the influencing mechanism to improve the SB of NGCWs with a specific focus on JS, SKS and WE. The results carried out by empirical analysis can be summarized as five key findings as follows:

- The JS significantly and positively affects the SKS and WE of NGCWs with the influence effect of 0.849 and 0.945 based on H2 and H5, respectively;
- The SKS is positively related to the SCB and SPB of NGCWs, and the influence effect values are 0.599 and 0.687 based on H3a and H3b, respectively;
- WE has a significant positive effect on the SPB of NGCWs and the influence effect is 0.434 based on H6b;
- WE can play a crucial mediating role between the influence of JS on SPB of NGCWs with the indirect influence effect of 0.410 based on H7b;
- The SKS mediates the effects of JS on both the SPB and SCB of NGCWs with the indirect influence effect of 0.583 and 0.509 based on H4a and H4b, respectively.

These findings reflect that JS is a proper predictor of WE and SKS directly, and SB indirectly, of NGCWs, that is JS, as a positive emotion, can indeed promote ones’ in-role and extra-role behaviors [43] like WE, SKS and SB, which are proven in this research. Moreover, in the influence relationship of JS on SPB, SKS plays a greater mediating role than WE does, which makes construction companies pay more attention to building a proper knowledge sharing culture to enhance the NGCWs’ willingness to participate in safety-related activities. In addition, improving the SPB of NGCWs is the more effective way of carrying out safety management compared to SCB. This finding can be evidence that the traditional safety management which emphasizes adherence to regulations and procedures does not effectively work on the NGCWs because they are more sensitive to hard rules and more willing to obtain safety knowledge through a comfortable and soft atmosphere.

### 6.2. Theoretical Implications

It is widely accepted that the SB of construction workers is the crucial factor in safety management and this has been paid much attention to in previous studies [5]. The prior studies primarily focused on leadership behavior [111,112], safety culture and climate [113,114], safety attitudes [115,116] and safety norms [112,117,118]. However, limited consideration is paid to the importance of the reaction about an individual’s subject emotion to work like job satisfaction, especially in the construction industry. Moreover, younger migrant workers, resulting from the population adjustment in the process of China’s modernization [8,9], need more attention to be paid. This study proposed to improve the SB from the perspective of JS along with the specific characteristics of the NGCWs, and used WE and SKS as mediating variables to construct a theoretical influencing mechanism, and in addition, through the empirical study, verified the relationship between JS, SKS/WE and SPB, and also the relationship between JS, SKS and SCB.

Theoretically, this study enriches previous research in various ways. First, it verifies the positive effect of JS on SB, deepening the understanding of JS’s support on employees’ behavior [27,43,44]. Second, as for safety management in construction industry, what is consistent with prior studies is that JS has a great influence on the safety performance of construction workers [12,28]. This study furthermore develops the thought of Idress et al. [12] and Stoilkovska et al. [28], proving that JS enhances the SB of construction workers both in SPB and SCB. Third, this study expands the knowledge body of safety knowledge sharing, confirming the positive effect between SKS and SB, which is a grounded response to Nesheim and Gressgård [60]. Fourth, this study addresses the importance of WE as a mediating variable of JS and SB, innovatively brings WE into safety management in construction industry and provides evidence of WE’s positive impact. 

In addition, this research is targeting on the newly emerging group, the NGCWs, concerned with improving their SB. Previous studies on NGCWs mainly concentrates on the psychological intergenerational comparisons [119,120] and little research has been done on their SB and the influencing mechanism of SB either. This study expands the knowledge body of the literature on younger construction workers and provides evidence that the SB of NGCWs can be promoted by the improvement of JS and in which WE and SKS play important mediating roles. The SPB of NGCWs can be more easily promoted by the improvement of JS compared to SCB, which can be explained by their special characteristic of pro-social behavior [121].

### 6.3. Practical Implications

This study has some implications for construction companies. First, the SB of NGCWs is crucial in safety management as they are gradually becoming the main force in the labor market of the construction industry, and they may behave differently in safety-related activities because of group characteristics caused by intergenerational differences. To enhance this, the present study provides suggestions for improving the JS, SKS and WE of NGCWs. Second, while considering the key role of JS in the influencing mechanism, project managers should pay more attention to improve the JS of NGCWs by enhancing the SP, SC, SS, SE and SW. To achieve this target, the legal rights of workers need to be protected. Moreover, a competitive salary system and a healthy working environment are necessary. Third, the WE and SKS can be regarded as a reward or exchange for their satisfaction obtained from the organization, so this psychological contract should be fulfilled by promoting the level of JS about NGCWs. Fourth, NGCWs with high engagement levels are more likely to obey the safety regulations at work, so construction companies should create good organizational culture, establish a professional development-oriented training system and cultivate correct work values to enhance their engagement. At last, the importance of SKS should be fully understood, effective communication channels, knowledge sharing platforms and incentive mechanisms should be established to create a good SKS culture circumstance.

### 6.4. Limitations and Future Work

The limitations of this study can be summarized in the following four aspects. First, the survey data of this study come from a specific area of China in some certain crafts, thus the samples may not be overall representative, and the results are based on the analysis of 368 samples, which is a relatively small sample size. Future studies could expand the sample size of different areas and different types of projects to improve the generalizability of the findings, further studying the influence of JS on SB through WE and SKS. If the extension research that refers to the age group from 40 to 65, the way to collect data will remain the same to in order to guarantee the accurate comparisons to be made between two different age groups. Secondly, this study used WE and SKS as mediating variables to investigate the influence of JS on SB. Future studies could concentrate on other mediating variables like safety climate [82,122] and safety-culture attitudes [123] to fulfil the influencing mechanism; in addition, some ergonomic factors [124] that affect safety behavior could be considered in the mechanism to further expand the knowledge body of safety behavior in construction industry, which maybe provides a new perspective to explore the relationship of job satisfaction and construction workers’ safety behavior through both psychosocial and ergonomics factors. Third, due to the cross-sectional peculiarity of the questionnaire survey, the results concluded from obtained data at a given time, space and population only represent the relationship among variables within a short specific interval for the NGCWs. Research within a relatively long interval could be carried out by future studies and compare with the results in this study. Fourth, this study was conducted through self-report questionnaires, and the interference of “social expectation response” may cause some inaccuracy. Therefore, more behavioral measures should be considered for assessing the NGCWs’ JS, WE and SB or experimental research can be carried out in this area. 

## 7. Conclusions

With the NGCWs born after 1980 gradually taking the place of older labor in the construction industry of China, their safety behaviors need more attention to be paid. Traditional safety management strategies, like penalties, may not work on NGCWs because of their desire for higher interests and more humanistic care from the organization. Given the circumstance of job satisfaction’s positive impact on job performance, knowledge sharing behavior, workplace productivity and work engagement, it attracts much concern in the construction industry. However, none of the research has conducted to a link between job satisfaction and safety behavior. To fully understand the influencing mechanism, this study focuses on the effect of job satisfaction on safety behavior using the mediating roles of work engagement and safety knowledge sharing in linking the job satisfaction of safety behavior about NGCWs, the conclusions can be drawn as follows:

(i) Job satisfaction significantly and positively affects the safety knowledge sharing and work engagement of NGCWs. This verifies the importance for the construction companies to improve employees’ JS to make them more willing to share safety knowledge and work at a high level of vigor.

(ii) Safety knowledge sharing is positively related to the safety compliance behavior and safety participation behavior of NGCWs. This conclusion shows that safety knowledge sharing does have a positive impact on the safety work conduct of NGCWs and it is necessary to establish an appropriate circumstance for safety knowledge sharing in order to promote safety outcomes. 

(iii) Work engagement has a significant positive effect on the safety participation behavior of NGCWs. This conclusion shows that the higher level of engagement, the more willingness of NGCWs will have in participating in safety-related activities.

(iv) Work engagement plays a crucial mediating role between the influence of JS on the safety participation behavior of NGCWs, meanwhile, safety knowledge sharing mediates the effects of JS on both the safety participation behavior and safety compliance behavior of NGCWs, which expands the knowledge body of SB and gives examples to construction companies that it is a more effective way to encourage employees to exchange experience and information carrying valuable knowledge for their compliance and participation in safety-related activities.

In conclusion, few studies have conducted specific investigations on NGCWs, who are gradually becoming the major labor force in China’s construction industry. To fill this research gap, this paper explored the mechanism of improving safety behavior from the perspective of the job satisfaction of NGCWs, and used work engagement and safety knowledge sharing as two moderating variables to establish a theoretical model. The hypotheses results show that safety knowledge sharing plays a mediation role in the relationship between job satisfaction and safety participation behavior and safety compliance behavior, and work engagement mediates the relationship between job satisfaction and safety participation behavior. 

From a practical stand point, an excellent social security system and salary system, human-based work environment and attention to the psychological contract of employees in the construction company may help with the job satisfaction of NGCWs. Their work engagement can be improved by building great organizational culture, establishing a career-oriented training system and cultivating positive work value concepts. Effective communication channels, knowledge sharing culture and an incentive system might promote the NGCWs’ safety knowledge sharing.

## Figures and Tables

**Table 1:** Demographic information of respondents (*N* = 368).

Variable	Categories	Number of Cases	Frequency (%)
Sex	Male	343	93.2
	Female	25	6.8
Age	Between 16 and 20	7	1.9
	Between 21 and 30	126	34.2
	Between 31 and 40	235	63.9
Marital Status	Unmarried	64	17.4
	Married	304	82.6
Educational Level	Primary school and below	11	3.0
	Junior high school	144	39.1
	Certificate or associate’s degree	107	29.1
	Senior high school	71	19.3
	Junior college and above	35	9.5
Seniority	Less than 6 years	106	28.8
	Between 6 and 10 years	178	48.4
	Between 11 and 15 years	61	16.6
	Between 16 and 20 years	17	4.6
	Greater than 20 years	6	1.6
Working hours in a day	Less than 8 h	16	4.3
	Between 8 and 10hours	303	82.3
	Greater than 10 h	49	13.3
Average monthly income	less than USD 300	5	1.4
	USD 300–450	7	1.9
	USD 450–600	28	7.6
	USD 600–750	43	11.7
	USD 750–900	76	20.7
	USD 900–1050	83	22.6
	USD 1050–1200	60	16.3
	USD 1200 and above	66	17.9

**Table 2:** Construct validity and reliability (*N* = 368).

Variable	Construct	Item	Cronbach’s Alpha	FL	CR	AVE
JS	SP	SP1	0.871	0.784	0.879	0.594
		SP2		0.780		
		SP3		0.820		
		SP4		0.813		
		SP5		0.644		
	SC	SC1	0.900	0.823	0.889	0.618
		SC2		0.806		
		SC3		0.860		
		SC4		0.642		
		SC5		0.782		
	SL	SL1	0.919	0.812	0.920	0.696
		SL2		0.807		
		SL3		0.832		
		SL4		0.854		
		SL5		0.865		
	SE	SE1	0.904	0.749	0.896	0.685
		SE2		0.805		
		SE3		0.847		
		SE4		0.896		
	SW	SW1	0.885	0.772	0.888	0.613
		SW2		0.804		
		SW3		0.750		
		SW4		0.832		
		SW5		0.752		
SKS	ESKS	ESKS1	0.941	0.851	0.940	0.759
		ESKS2		0.874		
		ESKS3		0.899		
		ESKS4		0.896		
		ESKS5		0.834		
	TSKS	TSKS1	0.947	0.903	0.947	0.780
		TSKS2		0.901		
		TSKS3		0.899		
		TSKS4		0.877		
		TSKS5		0.835		
WE	JI	JI1	0.897	0.828	0.894	0.629
		JI2		0.869		
		JI3		0.765		
		JI4		0.725		
		JI5		0.770		
	OI	OI1	0.934	0.886	0.931	0.729
		OI2		0.905		
		OI3		0.852		
		OI4		0.818		
		OI5		0.805		
	WV	WV1	0.932	0.726	0.932	0.733
		WV2		0.824		
		WV3		0.901		
		WV4		0.907		
		WV5		0.909		
SB	SCB	SCB1	0.915	0.814	0.914	0.641
		SCB2		0.850		
		SCB3		0.853		
		SCB4		0.707		
		SCB5		0.745		
		SCB6		0.823		
	SPB	SPB1	0.947	0.849	0.946	0.744
		SPB2		0.875		
		SPB3		0.877		
		SPB4		0.869		
		SPB5		0.859		
		SPB6		0.847		

Note: JS = job satisfaction; SP = satisfaction with pay; SC = satisfaction with co-workers; SL = satisfaction with leaders; SE = satisfaction with the work environment; SW = satisfaction with work itself; SKS = safety knowledge sharing; ESKS = explicit safety knowledge sharing; TSKS = tacit safety knowledge sharing; WE = work engagement; JI = job involvement; OI = organizational identification; WV = work value; SB = safety behavior; SCB = safety compliance behavior; SPB = safety participation behavior; FL = factor loading; CR = composite reliability; AVE = average variance extracted.

**Table 3:** Descriptive statistics and correlation analysis (*N* = 368).

Variable	Mean	SD	SP	SC	SL	SE	SW	ESS	ISS	JI	OI	WV	SCB	SPB
SP	3.120	0.847	***0.771***											
SC	3.836	0.770	0.543 **	***0.786***										
SL	3.617	0.878	0.641 **	0.744 **	***0.834***									
SE	3.402	0.957	0.714 **	0.593 **	0.743 **	***0.828***								
SW	3.516	0.848	0.657 **	0.646 **	0.724 **	0.731 **	***0.783***							
ESS	3.673	0.904	0.534 **	0.611**	0.652 **	0.606 **	0.701 **	***0.871***						
ISS	3.734	0.881	0.624 **	0.624 **	0.667 **	0.616 **	0.721 **	0.904 **	***0.883***					
JI	3.676	0.818	0.557 **	0.675 **	0.718 **	0.661 **	0.815 **	0.722 **	0.746 **	***0.793***				
OI	3.578	0.900	0.609 **	0.669 **	0.708 **	0.695 **	0.787 **	0.743 **	0.779 **	0.836 **	***0.854***			
WV	3.629	0.892	0.560 **	0.630 **	0.691 **	0.668 **	0.786 **	0.737 **	0.765 **	0.826 **	0.887 **	***0.856***		
SCB	4.051	0.795	0.388 **	0.604 **	0.566 **	0.512 **	0.556 **	0.678 **	0.694 **	0.636 **	0.636 **	0.632 **	***0.801***	
SPB	3.946	0.869	0.420 **	0.603 **	0.597 **	0.546 **	0.632 **	0.751 **	0.786 **	0.685 **	0.693 **	0.714 **	0.837 **	***0.863***

Note: the italic and bolded numbers are the square roots of AVE. **, *p* < 0.01.

**Table 4:** Descriptive statistics and correlation analysis (*N* = 368).

Fit Index	Scores	Recommended Cut-Off Value
Absolute fit measures		
χ^2^/df	2.248	≤2 ^a^; ≤5 ^b^
RMR	0.053	≤0.05
GFI	0.741	≥0.9 ^a^, ≥0.8 ^b^
RMSEA	0.058	<0.08 ^a^; <0.1 ^b^
Incremental fit measures		
NFI	0.842	≥0.9 ^a^, ≥0.8 ^b^
IFI	0.906	≥0.9
TLI/NNFI	0.901	≥0.9
AGFI	0.718	≥0.9 ^a^, ≥0.8 ^b^
CFI	0.906	≥0.9
Parsimonious fit measures		
PGFI	0.681	≥0.5, the higher, the better
PNFI	0.801	≥0.5, the higher, the better
PCFI	0.861	≥0.5, the higher, the better

Note: ^a^, equals acceptable. ^b^, equals marginal; RMR = root-mean square residual; GFI = goodness-of-fit index; RMSEA = root mean square error of approximation; NFI = normed fit index; IFI = incremental fit index; TLI = tacker-lew index; NNFI = non-normed fit index; AGFI = adjusted goodness-of-fit index; CFI = comparative fit index; PGFI = parsimony goodness-of-fit index; PNFI = parsimony normed fit index and PCFI = parsimony comparative fit index.

**Table 5:** Hypothesis testing results (*N* = 368).

Hypothesis	Path	Path Coefficient	C.R.	*p*	Result
H1a	JS–SCB	−0.015	−0.070	0.944	Not supported
H1b	JS–SPB	−0.247	−1.360	0.174	Not supported
H2	JS–SKS	0.849	12.418	***	Supported
H3a	SKS–SCB	0.599	6.434	***	Supported
H3b	SKS–SPB	0.687	8.675	***	Supported
H5	JS–WE	0.945	12.533	***	Supported
H6a	WE–SCB	0.194	1.054	0.292	Not supported
H6b	WE–SPB	0.434	2.809	0.005	Supported
H7a	JS–WE–SCB	——	——	——	Not supported

Note: ***, *p* < 0.001; C.R. = critical ratio.

**Table 6:** Total indirect effect test.

Path	Bootstrapping				Total Indirect Effect
	Bias-Corrected 95% CI		Percentile 95% CI		
	Lower	Upper	Lower	Upper	
JS→SCB	0.580	0.752	0.585	0.755	Exist
JS→SPB	0.657	0.808	0.659	0.811	Exist

Note: CI = confidence interval.

**Table 7:** Specific indirect effect test.

Hypothesis	Path a		Path b		Specific Indirect Effect	Total Indirect Effect	CI (95%)		Result
	Path Coefficient	Standard Error	Path Coefficient	Standard Error			Lower	Upper	
H4a:JS–SKS–SPB	0.849	0.030	0.687	0.082	0.583	0.993	0.416	0.769	Supported
H4b:JS–SKS–SCB	0.849	0.030	0.599	0.096	0.509	0.692	0.418	0.602	Supported
H7b:JS–WE–SPB	0.945	0.015	0.434	0.196	0.410	0.993	0.046	0.797	Supported

Note: path a represents the effect of an independent variable on the proposed mediator; path b represents the effect of the proposed mediator on dependent variable partialing out the effect of independent variable; CI = confidence interval.

## Appendix A

**Table A1:** Questionnaire items in measurement scales.

Construct	Code	Measurement Item
SP	SP1	My organization pays better than competitors.
	SP2	My pay is adequate, considering the responsibilities I take.
	SP3	I am sufficiently paid for what I do.
	SP4	My fringe benefits are generous.
	SP5	My salary can be paid in time as agreed.
SC	SC1	When I ask people to do things, the job gets done.
	SC2	I enjoy working with the people here.
	SC3	I work with responsible people.
	SC4	There is little bickering and fighting at work.
	SC5	It is easy to do teamwork with my co-workers.
SL	SL1	The managers I work for back me up.
	SL2	The managers I work for are “topnotch”.
	SL3	My leaders listen to me.
	SL4	My leaders treat me fairly.
	SL5	I am in favor of the way my leaders handle his/her workers.
SW	SW1	My job is interesting.
	SW2	I feel my job meaningful.
	SW3	I don’t intent to job-hop at present.
	SW4	I feel a sense of pride in doing my job.
	SW5	I can bear the labor intensity every day.
SE	SE1	I am quite satisfied with the food conditions.
	SE2	I am quite satisfied with the accommodation conditions.
	SE3	I am quite satisfied with the safety protection measures.
	SE4	I am quite satisfied with the working environment.
JI	JI1	At my job, I am very resilient, mentally.
	JI2	At my job I feel strong and vigorous.
	JI3	At my work I always persevere, even when things do not go well.
	JI4	I am immersed in my work.
	JI5	Time flies when I am working.
WV	WV1	To me, my job is challenging.
	WV2	My job inspires me.
	WV3	I am enthusiastic about my job.
	WV4	I am proud of the work I do.
	WV5	I find the work I do full of meaning and purpose.
OI	OI1	Being a member of this organization is exhilarating for me.
	OI2	I care whether I can contribute to the company or the team.
	OI3	I will introduce the benefits of working here to friends and family.
	OI4	I would like to recommend my company to my friends who are looking for a job.
	OI5	It is exciting for me to get involved in things happening in the organization.
ESKS	ESKS1	I share my work reports and official documents with members of my organization frequently.
	ESKS2	I always provide my manuals, methodologies and models for members of my organization.
	ESKS3	I often discuss and exchange the contents of daily safety meetings with my workmates.
	ESKS4	I often discuss and exchange safety training contents with my colleagues.
	ESKS5	I often discuss and exchange construction safety accident news with my workmates.
TSKS	TSKS1	I share my experience or know-how from work with other organizational members.
	TSKS2	I share my expertise from my education or training with other organizational members.
	TSKS3	People in my organization will share lessons from past failures when they feel necessary.
	TSKS4	I talk about my tips on jobs with my co-workers.
	TSKS5	My workmates and I often observe and imitate each other in our work.
SCB	SCB1	I always wear a safety helmet during operations at my job site.
	SCB2	I use the correct safety procedures for carrying out my job.
	SCB3	I ensure the highest levels of safety when I carry out my job.
	SCB4	I will not try to do construction work that I am not familiar with.
	SCB5	I will not despite correct and safe work procedures due to over familiarity with the job.
	SCB6	I do not skip safety procedures even if under pressure to complete a job as soon as possible.
SPB	SPB1	I promote the safety program within the organization.
	SPB2	I put in extra effort to improve the safety of the workplace.
	SPB3	I voluntarily carry out tasks or activities that help to improve workplace safety.
	SPB4	I help my coworkers when they are working under risky or hazardous conditions.
	SPB5	I always point out to the management if any safety related matters are noticed in my company.
	SPB6	I willingly propose ideas to secure job site safety.
